# Supplementary material for: Two Ume6 target gene classes in the biofilm regulatory network of Candida albicans
Source: bioRxiv. 2026 Sep 24:2026.09.24.753443. Preprint. [Version 1] doi: 10.64898/2026.09.24.753443 (PMC13622266; doi:10.64898/2026.09.24.753443)
Supplement: Supplementary file 1 [file NIHPP2026.09.24.753443v1-supplement-4.pdf]

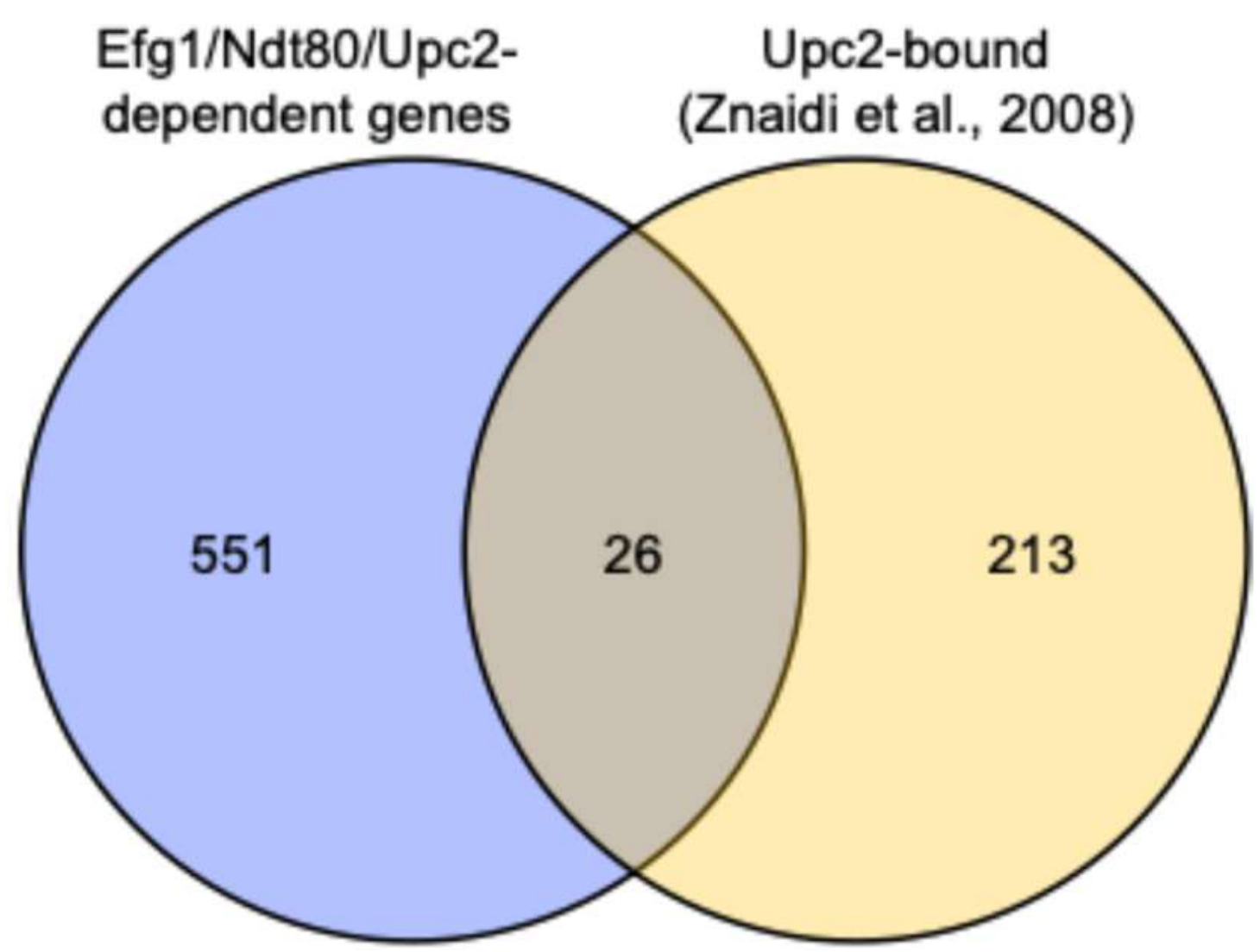

Fig S1. Upc2-bound genes in the Efg1/Ndt80/Upc2-dependent genes.
Venn diagram depicts intersection of the Efg1/Ndt80/Upc2-dependent genes and the Upc2-bound genes (Znaidi et al., 2008).

Motif	P-value	% of Targets	% of Background
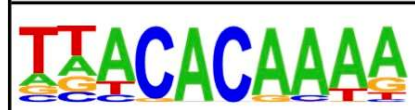	1.00E-40	35.97%	11.85%
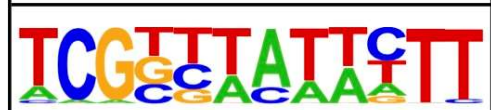	1.00E-37	21.20%	4.48%
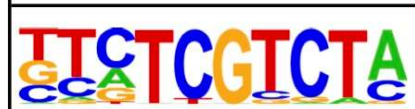	1.00E-30	17.13%	3.52%
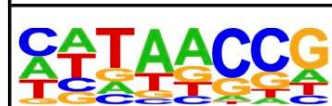	1.00E-17	49.46%	30.02%
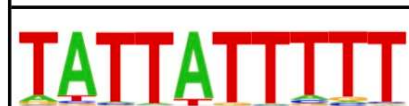	1.00E-16	48.82%	30.16%
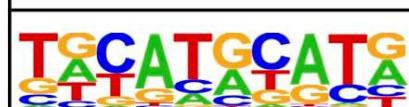	1.00E-15	13.28%	4.02%
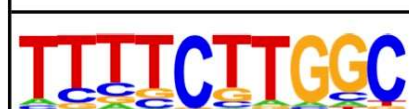	1.00E-12	21.63%	10.26%

Fig S2. Enriched binding motifs in the Efg1/Ndt80/Upc2-dependent genes.
De novo binding motif analysis for the 577 gene promoters was performed with HOMER.

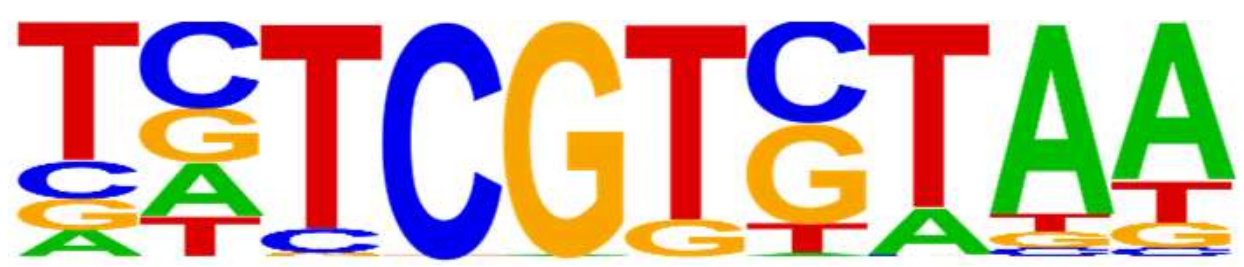

P-value: 1e-76
% of Targets: 45.70%
% of Background: 14.22%

Fig S3. Enriched binding motif in the Efg1/Ndt80/Upc2-independent genes.
De novo binding motif analysis for the 534 gene promoters was performed with HOMER.
