# Supplementary material for: Two Ume6 target gene classes in the biofilm regulatory network of Candida albicans
Source: bioRxiv. 2026 Sep 24:2026.09.24.753443. Preprint. [Version 1] doi: 10.64898/2026.09.24.753443 (PMC13622266; doi:10.64898/2026.09.24.753443)
Supplement: Supplementary file 2 [file media-3.pdf]

Strains and media

Strains used in this study were maintained in 15% glycerol frozen stocks at -80°C. Prior to use, cells were routinely grown on YPD agar plates (2% dextrose, 2% Bacto peptone, 1% yeast extract, 2% Bacto agar) for overnight at 30°C and then cultured in liquid YPD medium overnight at 30°C with agitation. Transformants were selected on YPD plus 400 µg/mL nourseothricin (clonNAT; Gold Biotechnology) or complete synthetic medium (2% dextrose, 1.7% Difco yeast nitrogen base with ammonium sulfate and auxotrophic supplements). All strains used in this study are listed in Table S1 in the supplemental material.

Strain constructions

To manipulate *C. albicans* genome, the transient CRISPR-Cas9 system was employed as previously described in detail (1). Generally, the Cas9 cassette was amplified from the plasmid pV1093, and each of sgRNA cassette was generated by using split-joint PCR with "sgRNA/F" and "SNR52/R" as previously described in detail (1, 2). Primers and plasmids used for transformation are listed in Table S1.

To construct a *efg1*Δ/Δ *ndt80*Δ/Δ *upc2*Δ/Δ triple mutant strain in the *efg1*Δ/Δ *ndt80*Δ/Δ double mutant background, the *UPC2* deletion cassette was amplified from the plasmid pSFS2A-CaKan with primers 'Upc2_dc_Kan/F' and 'Upc2_dc_Kan/R' (3). Transformants were screened on YPD containing 600 µg/ml G418 and 1.75 mg/ml molybdate, and candidates were genotyped by PCR using primers 'Upc2 check up/F' and 'Upc2 check int/R' for absence of *UPC2* ORF and using primers 'Upc2 check up/F' and 'Kan int/R' for the presence of the *CaKAN* marker at the *UPC2* locus.

To construct *UME6* overexpression strains (P_{RBT5}-*UME6-HA*) in both the *efg1*Δ/Δ *ndt80*Δ/Δ double and the *efg1*Δ/Δ *ndt80*Δ/Δ *upc2*Δ/Δ triple mutant strains, the *NAT1*-P_{RBT5} repair template and sgRNA cassette were prepared as previously described in detail (4). Then, the *UME6-3xHA-ADH1* terminator cassette was amplified from the plasmid "pSN52-HA-ADH1term" with primers "Ume6_F-HA" and "ADH1term -> HygB up/R". The *CaHygB* cassette was amplified from the plasmid "pSFS2A-CaHygB" with primers "Kan_HygB/F" and "HygB -> Ume6 down/R". Transformants were screened on YPD containing 600 µg/ml hygromycine B and 1.75 mg/ml quinine, and candidates were genotyped by PCR using primers 'Ume6-tag confirm F' and 'Ume6-tag confirm R' for absence of Ume6 terminator sequence and using primers 'Kan_Hygb/F' and 'Ume6_3UTR_R' for the presence of the *CaHYGB* marker at the *UME6* locus.

ChIP-seq and Bioinformatic analysis

ChIP samples and ChIP-seq libraries were prepared according to previously described methods (5). Bioinformatic analysis used in this study was performed according to previously described methods (5). Differential binding analysis (DiffBind, Galaxy Version 2.10.0+galaxy0) (6), de novo motif discovery (HOMER, v 4.11) (7), ComputeMatrix (Galaxy Version 3.5.1.0.0), and PlotHeatmap (Galaxy Version 3.5.4+galaxy0) were conducted according to previously described methods (8). Gene Ontology (GO) term enrichment analysis was determined with the GO Termfinder tool at the *Candida* Genome Database (9).

Data analysis software

ChIP-seq data were visualized using the Integrative Genomics Viewer v2.17.4 (10). Biofilm and filamentation images were processed using Image J (Fiji) (11). Statistical analyses and graph generations were carried out using GraphPad Prism version 9 (GraphPad Software, Inc., La Jolla).

Data availability

Processed ChIP-seq data is available in Supplementary Tables 2; raw data is available through NCBI SRA with accession numbers PRJNA1522041. Strains are available upon request.

References

1. Min K, Ichikawa Y, Woolford CA, Mitchell AP. 2016. Candida albicans Gene Deletion with a Transient CRISPR-Cas9 System. mSphere 1.
2. Huang MY, Woolford CA, May G, McManus CJ, Mitchell AP. 2019. Circuit diversification in a biofilm regulatory network. PLoS Pathog 15:e1007787.
3. Park SO, Frazer C, Bennett RJ. 2022. An Adjuvant-Based Approach Enables the Use of Dominant HYG and KAN Selectable Markers in Candida albicans. mSphere 7:e0034722.
4. Mao Y, Solis NV, Sharma A, Cravener MV, Filler SG, Mitchell AP. 2022. Use of the Iron-Responsive RBT5 Promoter for Regulated Expression in Candida albicans. mSphere 7:e0030522.
5. Do E, Cravener MV, Huang MY, May G, McManus CJ, Mitchell AP. 2022. Collaboration between Antagonistic Cell Type Regulators Governs Natural Variation in the Candida albicans Biofilm and Hyphal Gene Expression Network. mBio 13:e0193722.
6. Ross-Innes CS, Stark R, Teschendorff AE, Holmes KA, Ali HR, Dunning MJ, Brown GD, Gojis O, Ellis IO, Green AR, Ali S, Chin SF, Palmieri C, Caldas C, Carroll JS. 2012. Differential oestrogen receptor binding is associated with clinical outcome in breast cancer. Nature 481:389–93.
7. Heinz S, Benner C, Spann N, Bertolino E, Lin YC, Laslo P, Cheng JX, Murre C, Singh H, Glass CK. 2010. Simple combinations of lineage-determining transcription factors prime cis-regulatory elements required for macrophage and B cell identities. Mol Cell 38:576–89.
8. Do E, Zarnowski R, Andes DR, Mitchell AP. 2026. An emergent biofilm program from inactivation of Candida albicans master regulators Efg1 and Ndt80. PLoS Pathog 22:e1014469.
9. Lew-Smith J, Binkley J, Sherlock G. 2025. The Candida Genome Database: annotation and visualization updates. Genetics 229.
10. Robinson JT, Thorvaldsdottir H, Winckler W, Guttman M, Lander ES, Getz G, Mesirov JP. 2011. Integrative genomics viewer. Nat Biotechnol 29:24–6.
11. Schindelin J, Arganda-Carreras I, Frise E, Kaynig V, Longair M, Pietzsch T, Preibisch S, Rueden C, Saalfeld S, Schmid B, Tinevez JY, White DJ, Hartenstein V, Eliceiri K, Tomancak P, Cardona A. 2012. Fiji: an open-source platform for biological-image analysis. Nat Methods 9:676–82.
